# Association of liver enzymes with incident type 2 diabetes: A nested case control study in an Iranian population

**DOI:** 10.1186/1472-6823-8-5

**Published:** 2008-06-05

**Authors:** Maryam Tohidi, Hadi Harati, Farzad Hadaegh, Yadolladh Mehrabi, Fereidoun Azizi

**Affiliations:** 1 Prevention of Metabolic Disorders Research Center, Research Institute for Endocrine Sciences, Shahid Beheshti University (M.C), Tehran, Iran; 2 School of Public Health, Shahid Beheshti University (M.C), Tehran, Iran

## Abstract

**Background:**

To investigate the association of Aspartate aminotransferase (AST), Alanin aminotranferase (ALT) and Gamma glutamyl transferase (GGT) with incident type 2 diabetes.

**Methods:**

In a nested case-control study, AST, ALT, GGT as well as classic diabetes risk factors, insulin and C-reactive protein (CRP) were measured in 133 non-diabetic subjects at baseline of which 68 were cases and 65 were controls. Incident diabetes was defined by the WHO 1999 criteria. Conditional logistic regression was used to calculate the odds ratio (OR) of incident diabetes associated with different hepatic markers. We used factor analysis for clustering of classic diabetes risk factors.

**Results:**

In Univariate analysis both ALT and GGT were associated with diabetes with ORs of 3.07(1.21–7.79) and 2.91(1.29–6.53) respectively. After adjustment for CRP and insulin, ALT and GGT were still predictive of incident diabetes. When the model was further adjusted for anthropometric, blood pressure and metabolic factors, only ALT was independently associated with diabetes [OR = 3.18 (1.02–9.86)]. No difference was found between the area under the receiver operating characteristic curves of the models with and without ALT (0.820 and 0.802 respectively, P = 0.4)

**Conclusion:**

ALT is associated with incident type 2 diabetes independent of classic risk factors. However, its addition to the classic risk factors does not improve the prediction of diabetes.

## Background

In Recent years Non-Alcoholic Fatty Liver Disease (NAFLD) has drawn much attention to itself as a pathogenic factor of insulin resistance and type 2 diabetes mellitus [1]. This idea is supported by several cross-sectional studies showing an association between NAFLD and prevalence of type 2 diabetes as well as features of the metabolic syndrome, including dyslipidemia and abdominal obesity, which highlight insulin resistance as an important feature of NAFLD [2-4].

Since circulating liver enzymes including aspartate aminotransferase (AST), alanine aminotransferase (ALT), and Gamma glutamyltransferase (GGT) are commonly elevated in asymptomatic patients with NAFLD [5,6], the prospective association between the hepatic markers and type 2 diabetes is expected, as has been reported in many longitudinal studies [7-12]. However, the results of these observations are variable. For example while most of the studies demonstrated that serum GGT predicted type 2 diabetes independent of common diabetes risk factors, a study in Pima Indians did not [9]. Moreover, some [9-11], but not all studies [8] have demonstrated independent and significant associations of ALT with incident type 2 diabetes mellitus.

We aimed to investigate whether baseline serum liver enzymes including AST, ALT and GGT were associated with incident type 2 diabetes in non-diabetic participants of the Tehran Lipid and Glucose Study (TLGS) independent of clinical and metabolic risk factors as well as insulin resistance and C-reactive protein (CRP) and to see if they improve the predictive utility of the classic risk factors for development of type 2 diabetes.

## Methods

### Subjects

This was a nested case-control study among participants of the TLGS. The TLGS is a longitudinal study in which more than 15000 residents of the Tehran district 13 aged more than 3 years were selected by cluster random sampling method in the first phase of the study between 1999 and 2001 and were followed every 3 years for occurrence of diabetes [13]. The current study involves 10368 individuals older than 20 years. Each participant was interviewed privately after signing a written consent and was asked about past history including cigarette smoking, prior diagnosis and family history of diabetes and taking of anti-diabetes medications. Physical examination including measurement of anthropometric variables and blood pressure as well as fasting plasma glucose (FPG) and lipids was performed in all participants. The 75-g oral glucose tolerance test (OGTT) was also performed in subjects who did not take anti-diabetes medications.

Individuals were eligible for the current study if they were non-diabetic in the first phase. Based on α = 0.05, β = 0.10 and the estimated difference in the mean values of AST, ALT and GGT between cases and controls [7], the sample size was calculated as 63 for each group. After mean follow up of 3.5 years there were 188 new cases of type 2 diabetes of which 80 individuals were randomly selected as cases. Diabetes was defined as FPG ≥ 7.0 and/or 2-hours glucose ≥ 11.1 mmol/l or taking of anti-diabetic medications. For each case subject, a control subject who had remained non-diabetic at the time of the follow-up examination of the case was selected from the baseline population after matching for age and sex. After exclusion of subjects with missing serum backup (n = 16, 7 cases and 9 controls) and outliers (> 3SD distribution of the log transformed CRP and hepatic enzymes, n = 11, 5 cases and 6 controls), finally 68 cases and 65 controls entered into the current study.

### Clinical and Laboratory examinations

Detailed description of the methods for measuring anthropometric variables including weight, height, waist circumference (WC) and hip circumference has been previously reported [13]. Body mass index (BMI) was calculated as weight in kilograms divided by the height in meters squared. For calculation of waist-to-hip ratio (WHR), WC was divided by hip circumference. Systolic and diastolic blood pressures (SBP and DBP respectively) were measured twice in a sitting position after 15 minutes rest and mean of the two measurements was considered as the participant's blood pressure.

A blood sample was drawn between 7:00 and 9:00 AM into vacutainer tubes from all study participants after 12–14 hours overnight fasting. For each participant, a 5 ml serum sample was stored at -70° and was used for the baseline measurement of inflammatory and hepatic markers. The blood analyses of FPG and 2-hours OGTT, after oral administration of 82.5 g glucose monohydrate (equivalent to 75 g glucose anhydrate) solution as well as lipid measurements were done at the TLGS research laboratory on the day of blood collection. Plasma fasting and 2-hour glucose were measured using an enzymatic colorimetric method with glucose oxidase. Triglycerides (TGs) were assayed using enzymatic colorimetric method with glycerol phosphate oxidase. HDL-cholesterol (HDL-C) was measured using enzymatic calorimetric method with cholesterol esterase and cholesterol oxidase after precipitation of the apolipoprotein B-containing lipoproteins with phosphotungestic acid. AST and ALT were analyzed by enzymatic photometry and GGT by enzymatic calorimetric methods and their intra- and inter assay coefficients of variation (CVs) were 2.8 and 3.8% for AST, 2.2 and 3.8% for ALT, and 2.9 and 3.0% for GGT respectively. These analyses were performed using commercial kits (Pars Azmon Inc., Tehran, Iran) and a Selectra 2 auto-analyzer (Vital Scientific, Spankeren, The Netherlands). The intra and inter-assay coefficients of variation (CV) were 2.2% for glucose. For HDL-C intra and inter-assay CVs were 0.5 and 2% respectively. We measured insulin with the immunoenzymometric (IEMA) assay (Monobind Inc., Costa Mesa, USA) and the intra- and inter-assay coefficients of variation (CVs) were 9.2 and 10.3% respectively. Homeostatic model assessment for insulin resistance (HOMA-IR) model was used to indirectly measure insulin resistance and was calculated as Fasting glucose (mmol/l) × insulin (μU/l)/22.5. CRP was measured using highly sensitive IEMA (hsCRP, Diagnostic Biochem, Ontario, Canada) with intra- and inter-assay CVs of 7.7 and 9.7% respectively. The IEMAs were performed by Sunrise ELISA reader (Tecan Co. Salzburg, Austria).

### Statistical analysis

Baseline characteristics of the subjects were presented as percent and mean ± SD or median with inter-quartile range in cases of skewed variables. To achieve normal distribution, insulin, CRP and hepatic markers were log transformed before analysis. Chi-square and independent sample T-tests were used for comparison of baseline characteristics including hepatic markers. Bivariate Pearson correlation was assessed between the hepatic markers and common diabetes risk factors and CRP. Conditional logistic regression analysis was used to calculate the odds ratio (OR) with 95% confidence interval of hepatic markers associated with incident diabetes. All the hepatic markers were entered into the model as continuous variables and the ORs calculated referring to the risk of diabetes per one SD increment in them. ORs were calculated first in a univariate model in which only the individual hepatic marker was entered and then in 3 other models where HOMA-IR, CRP and common diabetes risk factors were also entered.

To control the statistical biases, resulting from adjusting for too many variables, and collinearity in the logistic regression model [14] and to meet the desired outcome to variable ratio of 10 [15], we used factor analysis to reduce the number of classic diabetes risk factors to a smaller set of uncorrelated variables [16]. According to the Kaiser criterion [16], factors with eigen-values > 1 were retained in the extraction step. Varimax rotation was used and the resulting pattern was interpreted based on rotated factor loadings > 0.4. We reported the cumulative percentage of variance accounted for by the current and all preceding factors. Factor scores, were calculated and entered into models as continuous independent variables. Area under the receiver operating characteristic curve (AUC) of the logistic regression models was used to compare their predictive power for incident type 2 diabetes [17]. SPSS 11.5 and STATA 9.0 software packages were used for the analysis and P values less than 0.05 were considered as statistically significant.

## Results

There were 68 new cases with type 2 diabetes and 65 age and sex matched controls. Table 1 show that almost all of the measured baseline risk factors were significantly higher in case subjects than controls except smoking status which was comparable in cases and controls. Of particular interest, cases had higher level of insulin and CRP than control subjects (P = 0.01 and P = 0.001 respectively). AST levels were comparable between cases and controls (P = 0.2) but baseline ALT and GGT were significantly higher in those who developed diabetes (P = 0.01 and 0.005 respectively).

**Table 1:** Baseline characteristics of subjects who did and did not develope diabetes after 3.5 years of follow-up.

	Without diabetes (Controls) N = 68	With diabetes (Cases) N = 65	P value
Age (years)	47 ± 13	47 ± 12	-
Male sex (%)	43	43	-
Current and ex smokers (%)	16	28	0.1
Family history of diabetes (%)	11	42	<0.001
BMI (kg/m^2^)	26.8 ± 4.6	30.0 ± 4.3	<0.001
Waist (cm)	89 ± 12	97 ± 10	<0.001
Systolic blood pressure (mm/Hg)	118± 14	127± 19	0.001
Diastolic blood pressure (mm/Hg)	78± 10	82± 11	0.01
Fasting plasma glucose (mmol/l)	4.8± 0.4	5.4± 0.6	<0.001
2-hour plasma glucose (mmol/l)	5.5± 1.2	7.1± 1.9	<0.001
Triglycerides* (mmol/l)	1.6 (1.1–2.3)	2.0 (1.4–2.8)	0.001
HDL cholesterol (mmol/l)	1.1± 0.3	0.9± 0.2	0.01
Fasting serum insulin* (IU/ml)	12.4 (7.6–17.4)	14.7 (11.1–20.6)	0.01
Homeostatic model assessment for insulin resistance (HOMA-IR)¶	2.8± 1.4	3.8± 1.7	<0.001
hs-CRP* (mg/l)	0.7 (0.3–2.0)	1.3 (0.7–2.7)	0.001
γ-glutamyl transferase* (IU/L)	16.9 (13.5–22.9)	21.3 (16.4–33.5)	0.005
Alanin aminotranferase* (IU/L)	15.5 (11.0–20.5)	19.0 (15.0–26.2)	0.01
Aspartate aminotransferase* (IU/L)	19.5 (17.0–24.5)	21.0 (18.0–27.0)	0.2

*For variables with skewed distribution, median with inter-quartile range was presented. For all other continuous variables data were presented as mean ± SD.

¶ HOMA-IR was calculated as fasting glucose (mmol/l) × insulin (μU/l)/22.5

Correlations between hepatic markers and common diabetes risk factors are presented in table 2. Whereas AST was not significantly correlated with any risk factor, ALT and GGT showed moderate statistically significant positive correlation with WC, WHR, FPG, 2hPG and TGs. The magnitude of these correlations was generally stronger for GGT. GGT also showed a significant positive correlation with CRP.

**Table 2:** Pearson correlation between hepatic markers and baseline characteristics of the subjects.

	γ-glutamyl transferase	Alanin aminotranferase	Aspartate aminotransferase
Body mass index	0.113	0.014	-0.046
Waist circumference	0.300**	0.176	0.053
Waist-to-hip ratio	0.440**	0.276**	0.173
Systolic blood pressure	0.122	-0.062	-0.006
Diastolic blood pressure	0.168	0.022	0.104
Fasting plasma glucose	0.308**	0.200*	0.016
2-hours plasma glucose	0.108	0.312**	0.005
Fasting insulin	0.117	0.081	0.000
Triglycerides	0.263**	0.022	0.048
HDL-cholesterol	-0.129	0.005	0.134
C-reactive protein	0.159*	-0.084	0.013

All of the hepatic markers as well as fasting insulin, Triglycerides and C-reactive protein were log transformed before analysis.

*Correlation was significant at the 0.05 level (2-tailed).

**Correlation was significant at the 0.01 level (2-tailed).

Table 3 provides the result of the factor analysis among classic diabetes risk factors. 3 factors were interpreted as 1)Anthropometric factor including BMI, WC and WHR, 2)Blood pressure factor including SBP, DBP and 2hPG, and 3)metabolic factor including FPG, 2hPG, log TGs and HDL-c which together accounted for 71% of the total variance.

**Table 3:** Factor loadings resulting from factor analysis of classic diabetes risk factors.

	Factors		
	
	Anthropometric	Blood pressure	Metabolic
Systolic blood pressure	0.24	**0.83**	0.11
Diastolic blood pressure	0.18	**0.85**	0.06
Body mass index	**0.69**	0.37	0.15
Waist circumference	**0.93**	0.22	0.17
Waist-to-hip ratio	**0.81**	-0.00	0.17
Fasting glucose	0.23	0.15	**0.60**
2-hours glucose	0.00	**0.42**	**0.48**
Log triglycerides	0.12	0.22	**0.66**
HDL-cholesterol	-0.17	0.32	**-0.73**

Cumulative % of total variance	28.3	52.1	71.0

All of the hepatic markers as well as fasting insulin, Triglycerides and C-reactive protein were log transformed before analysis. *Correlation was significant at the 0.05 level (2-tailed). **Correlation was significant at the 0.01 level (2-tailed).

In univariate analysis, AST was not significantly associated with type 2 diabetes whereas ALT and GGT had significant ORs of 3.07(1.21–7.79) and 2.91(1.29–6.53) respectively (Table 4). After adjustment for HOMA-IR and hs-CRP, ALT [OR: 3.62(1.37–9.53)] and GGT [OR: 2.70(1.15–6.35)] were independently associated with type 2 diabetes. When the model was adjusted for anthropometric and blood pressure factors in addition to HOMA-IR and CRP, the association of ALT and GGT with type 2 diabetes remained significant (P = 0.02 and P = 0.01 respectively); however after further adjustment for the metabolic factor, only ALT was significantly associated with type 2 diabetes with OR of 3.18(1.02–9.86). Comparison of the AUC of the final model with and without ALT showed that addition of ALT did not significantly increase its predictive power [AUC = 0.846 (95% CI: 0.765–0.927) vs. 0.863(0.788–0.938), P = 0.2].

**Table 4:** Odds ratio of incident diabetes associated with hepatic markers in univariate models and after adjustment for other markers and common diabetes risk factors*.

	AST		ALT		GGT	
	
	Odds ratio	P	Odds ratio	P	Odds ratio	P
Model 1	1.96(0.64–5.95)	0.2	**3.07(1.21–7.79)**	**0.01**	**2.91(1.29–6.53)**	**0.01**
Model 2	1.27(0.47–3.42)	0.5	**3.62(1.37–9.53)**	**0.009**	**2.70(1.15–6.35)**	**0.02**
Model 3	1.56 (0.42–5.74)	0.5	**3.32(1.18–9.30)**	**0.02**	**3.23 (1.32–7.92)**	**0.01**
Model 4	1.09(0.34–3.53)	0.8	**3.18(1.02–9.86)**	**0.04**	1.93(0.73–5.04)	0.1

AST: Aspartate aminotransferase, ALT: Alanin aminotransferase, GGT: Gamma glutamyltransferase

*All the hepatic markers were log transformed before analysis. Odds ratios were calculated using backward conditional logistic regression with standardization to a SD of 1. Factor scores obtained from factor analysis were considered as continuous independent variables. In model 1 only the individual hepatic markers were entered (univariate). Model 2 was adjusted for log transformed C-reactive protein and HOMA-IR. Model 3 was further adjusted for family history of diabetes as well as anthropometric (waist circumference, waist-to-hip ratio and body mass index) and blood pressure (systolic and diastolic blood pressure and 2-hours glucose) factor scores. Model 4 was adjusted for all the variables in model 3 plus metabolic (Fasting and 2-hours glucose, triglycerides and HDL-cholesterol) factor score.

## Discussion

This is the first report in a Middle Eastern population which adds to the information regarding the role of hepatic markers in development of type 2 diabetes. It demonstrates that among hepatic markers only ALT is significantly associated with development of type 2 diabetes independent of classic risk factors as well as markers of whole body insulin resistance (HOMA-IR) and sub-clinical inflammation (CRP). However, addition of ALT to the previously known risk factors does not improve their predictive power for type 2 diabetes.

In the current study ALT and GGT showed significant correlation with WC, WHR, FPG 2hPG, and TGs which is consistent with the results of the studies that have shown a strong association between hepatic enzymes and various factors related to insulin resistance and the metabolic syndrome [2,3].

A number of prospective studies have investigated the association of liver markers with incident type 2 diabetes. Although most of these studies showed that GGT was an independent predictor of diabetes [7,8,12,18,19], few of them adjusted for the full range of classic diabetes risk factors [12] and none adjusted for baseline 2-hour glucose, a strong and consistent risk factor for type 2 diabetes. In a study in Pima Indians, in which AST, ALT and GGT were measured at baseline, only ALT predicted diabetes after adjustment for age, sex, percent body fat and insulin sensitivity [9]. In the west of Scotland coronary prevention study [10], high ALT but not AST predicted diabetes after adjustment for BMI, SBP, TC/HDL-C ratio, TGs and FPG. In the Insulin resistance Atherosclerosis study (IRAS) [11], both high ALT and AST were associated with incident diabetes after adjustment for full range of diabetes risk factors in addition to the markers of insulin sensitivity. On the other hand, in 3 recent population-based studies [7,20,21], ALT lost its association with incident diabetes after adjustment for either a minimum [7] or full range of diabetes risk factors [20,21]. One possible explanation for the variability of these observations may be explained both in terms of insufficient understanding of the biology of the liver enzymes and incomplete capture of their correlates and confounders [1]. Ethnicity could also have some role in this regard since separate analysis of the Hispanic and black subjects in the IRAS showed a non-significant association of liver markers with diabetes risk [11].

In the current study, GGT predicted diabetes after adjustment for family history of diabetes as well as anthropometric and blood pressure factors including BMI, WC, WHR, SBP and DBP; However it lost its association with diabetes after further adjustment for metabolic factors including FPG, 2hPG, TGs and HDL-C, which are some of the major components of the metabolic syndrome and might be directly related to liver fat [1]. In fact, elevated liver enzymes even within their normal range correlate well with increasing hepatic fat and NAFLD [22], which is in turn related to visceral fat deposition and general body insulin resistance [2]. However, the fact that in the current study both ALT and GGT were significantly associated with incident type 2 diabetes independent of markers of abdominal obesity and HOMA-IR (a sensitive marker of whole body insulin resistance) may highlight the role of hepatic insulin resistance and decline in hepatic insulin sensitivity in the association between hepatic markers, especially ALT, and incident type 2 diabetes [11].

It has been suggested that inflammatory markers, via their ability to enhance de novo hepatic fatty acid synthesis and fat accumulation, may contribute to both elevated liver enzymes and diabetes [9,23]. Significant correlation between GGT and CRP in the current study supports this finding. However, the fact that adjustment for CRP had minor effect on the association of GGT with incident type 2 diabetes may indicate that GGT might also be involved in the pathogenesis of type 2 diabetes through non-inflammatory mechanisms related to oxidative stress [24].

In the current study, ALT was predictive of diabetes after adjustment for all of the classic risk factors. However, it has been suggested that making decision about the predictive utility of a particular variable for diabetes risk would be more accurate if based on its additional utility over traditional risk factors rather than its independent association and relative risk [25]. Hence, the conclusion that hepatic enzymes may be useful additional measures in identifying the population at risk of diabetes, as several authors have said [10,26], must rely on appropriate statistical tests like calculation of AUC of the models with and without the particular variable. In this regards, the current study showed that ALT does not add to the predictive power of the model based on classic diabetes risk factors.

Our study has some limitations the first of which is small sample size. We compensated for this limitation by reducing the number of covariates in the logistic regression models by using factor analysis to justify the variable to outcome ratio and to reduce the statistical bias [14,15]. The second limitation is that we did not measure baseline markers of hepatitis B and C infection which considering the high prevalence of these infections in Iran [27], could have resulted in elevated liver enzymes. Alcohol consumption, a known cause of the elevated enzymes, is an unlikely confounder because of religious believes in the Iranian population. Definition of diabetes based on FPG and 2hPG measurement to identify undiagnosed cases, adjusting for all of the classic diabetes risk factors and using AUC as a measure of model discrimination are some of the strength of the current study.

The current study suggests that although serum ALT is a strong and independent predictor and might be involved in the pathogenesis of type 2 diabetes, its measurement in order to strengthen the predictive power of the classic risk factors for development of diabetes may not be justified.

## Pre-publication history

The pre-publication history for this paper can be accessed here:


